# Epidemiological profile of meningitis in Iran before pentavalent vaccine introduction

**DOI:** 10.1186/s12887-019-1741-y

**Published:** 2019-10-22

**Authors:** Zeinab Berangi, Manoochehr Karami, Younes Mohammadi, Milad Nazarzadeh, Seyed Mohsen Zahraei, Hamidreza Javidrad, Saber Heidari

**Affiliations:** 1 Department of Epidemiology, School of Public Health, Hamadan University of Medical Sciences, Hamadan, 65178-3-8736 Iran; 2 Research Center for Health Sciences, Hamadan University of Medical Sciences, Hamadan, Iran; 3 Social Determinants of Health Research Center, Hamadan University of Medical Sciences, Hamadan, Iran; 4 George Institute for Global Health, University of Oxford, Oxford, UK; 5 Center for Communicable Diseases Control, Ministry of Health & Medical Education, Tehran, Iran

**Keywords:** Public health surveillance, Meningitis, Iran, *Streptococcus pneumoniae*, *Neisseria meningitidis*, *Haemophilus influenzae*

## Abstract

**Background:**

No reliable and comprehensive study has been published on the incidence and epidemiological profile of meningitis in Iran from 2008 to 2014, before pneumococcal conjugate vaccine (PCV) and pentavalent vaccine (DTPw-Hep B-Hib (PRP-T) vaccine (pentavac) (adsorbed)) introduction. The present study aimed to portray the epidemiological profile of meningitis in Iran from 2008 to 2014.

**Methods:**

Data on meningitis cases aged from 1 day to 110 years were extracted from national notifiable diseases surveillance system from March 2008 to December 2014 in Iran. A total number of 48,006 cases of suspected meningitis were identified and 1468 cases of which met the criteria for diagnosis-confirmed meningitis. Of 1468 cases, 1352 patients were included in the study.

**Results:**

The great number of cases reported from urban areas. Moreover, males were more predominant than females (58.51% vs. 33.81%) in total. The estimated annual incidence rate of meningitis varied from 0.28/100000 in 2008 to 0.09/100000 in 2014*. Streptococcus pneumoniae, Haemophilus influenzae* and *Neisseria meningitidis* were the most leading pathogens causing bacterial meningitis, accounted for 266(23.44%), 145(12.78%), 95(8.37%) of cases, respectively. Each of the three bacterial species showed a descending trend. The majority of infected subjects are children under five years.

**Conclusions:**

Unlike the decreasing trend of meningitis and high percentage of cultures with negative results, according to World Health Organization recommendation PCV introduction into routine immunization is evident. Implementing an enhanced surveillance system to provide high quality data on epidemiological profile of meningitis in Iran is necessary.

## Background

Meningitis is defined as an inflammation of the meninges, the protective covering that surrounds the brain and spinal cord, mostly caused by bacterial, viral and fungal infection. Moreover, meningitis can be triggered by chemical irritation, subarachnoid hemorrhage, cancer and other conditions [1]. However, aseptic meningitis is more frequent compared to bacterial meningitis with a non-malignant clinical course [2]. The results of the studies done so far show that bacterial meningitis is a serious threat to global health in that there are 1.2 million cases of bacterial meningitis all over the world, accounting for almost 171,000 deaths annually [3, 4]. Although the incidence of meningitis is reported to be high among infants and children, it may be observed among healthy older children and adolescent. Despite the continuous advances in the development of more effective antibacterial medications every year, bacterial meningitis has a high fatality rate of 2 to 30% [5, 6]. Moreover, the neurological sequelae arise in about 30% of the survivors [7]. Ninety eight percent of 5.6 million disability-adjusted life years (DALYs) attributed to meningitis occur in countries with a low or middle income. In high-income countries, bacterial meningitis is among the top ten causes of death in children under the age of 14 [8]. *Streptococcus pneumoniae, Haemophilus influenzae and Neisseria meningitidis* the most leading organisms causing bacterial meningitis universally,are responsible for 90% of reported cases in childhood [9]. Subsequent to the development of highly effective conjugated vaccines to prevent meningitis universally, major variations in the epidemiological profile of meningitis are expected. Hence, the monitoring of disease trend in pre and post-vaccination era and the scrutiny of its epidemiological characteristics particularly those of bacterial meningitis seem necessary.

Meningitis is reported as a national notifiable diseases surveillance system in Iran. Such a system can provide detailed information concerning the geographical distribution of diseases, age and sex, groups at risk, the disease trend and the possible occurrence of epidemics. The information can be provided with the result that necessary preventive steps could be taken through designing and conducting research studies. These are to help specialists to design health interventions, and to evaluate effectiveness of implemented health interventions. Since national notifiable diseases surveillance system of meningitis has applied a passive approach. So, it is most likely to be underestimated the incidence of meningitis. Moreover pentavalent vaccine (DTPw-Hep B-Hib (PRP-T) vaccine (pentavac) (adsorbed)) was introduced in Iran in November 2014.

So far, there have been no reports regarding the incidence of meningitis in Iran, and the majority of the published studies associated with meningitis have been carried out on single etiological agent and certain geographical areas. Hence, the present study, using surveillance data aimed to portray the epidemiologic profile of meningitis in the country over a period of 7 years (from 2008 to 2014). It is expected that after the necessary precise information is gathered through evaluating the current situation in the country, significant steps can be taken towards making evidence-based decisions in order to manage and organize patients with meningitis so that the disease can be controlled and prevented.

## Methods

Data were extracted from the national notifiable diseases surveillance system from March 2008 to December 2014. The Center for Communicable Diseases Control in Iran has implemented a national surveillance system for meningitis. The current surveillance system in the country is a population-based surveillance therefore, all hospital of medical sciences are required to report all meningitis cases using passive approach [10–13]. In order to achieve the study objectives, the general information related to demographic variable (age, sex and location), para-clinical results, final diagnosis, outcome of patients, and vaccination status of patients were extracted using the standardized case report form.

In our set-up, we classified the patients into three case types (suspected, probable and confirmed cases). The following clinical and laboratory criteria were used as a basis for the definition of case types and the subsequent classification of the patients into 3 different groups and viral meningitis [14].:

### Suspected

Anyone at any age with a sudden onset of fever (38.5 °C rectal or 38 °C axillary) and at least one of the following signs observed

- Neck stiffness
- Bulging fontanel (for infant)
- Altered consciousness or other meningeal sign (including headache, vomiting and any type of sudden neurologic sequelae.

### Probable

A suspected case whose cerebrospinal fluid (CSF) examination shows at least one of the followings

- Turbid or purulent appearance
- Neutrophilic pleocytosis in CSF (≥100 white blood cells/mm3)
- Pleocytosis (10–100 white blood cells/mm3) along with increased protein (> 100 mg/dl) or hypoglycorrhachia
- A Gram stain results showing one of these findings
  -Gram negative coccobacilli (suggesting *Haemophilus influenzae*)
  -Gram positive diplococci (suggesting *Streptococcus pneumoniae*)
  -Gram negative diplococcic (suggesting *Neisseria meningitidis*)

### Confirmed

A suspected or probable cases with one of the followings

- Positive culture of CSF or blood with identification *Streptococcus pneumoniae, Haemophilus influenzae and Neisseria meningitidis* (gold standard for confirmed diagnosis) or
- Positive CSF antigen detection for *Streptococcus pneumoniae, Haemophilus influenzae and Neisseria meningitidis* by latex agglutination test (LAT) if available

Aseptic meningitis was defined as follows:

All of suspected cases of meningitis with negative/normal CSF, based on age, clinical symptoms of patients and cytological and biochemical parameters are considered as aseptic meningitis.

- Suspected viral meningitis: cases of fever (more than 38.5 degrees) and neck stiffness with changes in consciousness or prominent tangents and fontanels in infants and children.
- Probable Viral Meningitis: A suspected case with at least one of the following:
  -Normal glucose in the cerebrospinal fluid and a mild or normal increase in cerebrospinal fluid protein (< 50 mg / dl), a moderate increase in cerebrospinal fluid (< 500 cells / mm) and lymphocyte excretion (<% 50).
  -Epidemiological link with a definite case or existing epidemic
- Confirmed viral meningitis: a suspected or probable case with laboratory confirmation or negative culture of cerebrospinal fluid.

Finally, data were analyzed by Stata software, version 11 (Stata Corp, College Station, TX, USA) using descriptive statistics (frequency, mean, standard deviation). To investigate the association between qualitative variables the chi-squared test was used.

Incidence rate of meningitis of all types for each year of the study period was calculated by dividing the number of cases observed in each year by the average population of the country in the same year. For this purpose, the available data concerning the population of the country were obtained from the official website of the Statistical Center of Iran. In addition, the monthly trend of meningitis cases and the frequency distribution of bacterial meningitis cases according to pathogenic agent were plotted on line graphs. All statistical analysis were performed at a confidence interval (CI) of 95%.

## Results

During the study 48,006 patients (age ranging from 1 day to 110 years) with clinical sign and symptoms of meningitis were included in the study. Of the total 48,006 cases identified in this study, 9853 cases were categorized as probable while 1468 ones as confirmed meningitis, 116 cases of whom were excluded because of missing data. Of 1352 cases, bacterial meningitis was more prevalent as 1135 (77.31%) of cases is accounted for, followed by 217 (14.78%) of cases as aspetic meningitis. The percentage of male cases were 58.51, the percentage of female cases were 33.51. The sex distribution suggested that the incidence of bacterial meningitis in men is higher than women. A similar result was also reported for aseptic meningitis although it was not statistically significant (*p* = 0.232). In addition, the results of the statistical analyses showed that approximately 73% of patients with both aseptic and bacterial meningitis were resident in urban areas (*p* = 0.354). The present study demonstrated that the frequency of aseptic meningitis is more common in age group of under 5 years (62.21%) as 38% of which were seen in children under 1 year and 49.42% of bacterial origin were observed among children under 5 years (*p* < 0.001). *Streptococcus pneumoniae* was the prevailing microorganism that found in 266 (23.44%) cases [Table 1].

**Table 1:** The epidemiological and laboratory characteristics of patients with meningitis

Variable	Categories of variable	Bacterial meningitis *N*(%)	Aseptic meningitis *N*(%)	Total *N*(%)
Sex	Male	720 (63.44)	139 (46.06)	859 (58.51)
	Female	414 (36.48)	78 (35.94)	492 (33.51)
Residency Area	Urban	839 (73.92)	159 (73.27)	998 (67.98)
	Rural	283 (24.93)	55 (25.35)	338 (23.02)
	Nomads	2 (0.18)	–	2 (0.14)
	Mobile teams	5 (0.44)	2 (0.92)	7 (0.48)
Age	< 1 year	346 (30.48)	84 (38.71)	430 (29.29)
	1–5	215 (18.94)	51 (23.5)	266 (18.11)
	5	574 (50.57)	82 (37.79)	656 (44.68)
Etiological agents	*Streptococcus pneumoniae*	266 (23.44)	Unknown	266 (18.12)
	*Haemophilus influenzae* type b	145 (12.78)	Unknown	145 (9.88)
	*Neisseria meningitidis*	95 (8.37)	Unknown	95 (6.47)
	Other cases	629 (55.42)	Unknown	629 (42.85)
Treatment Outcome	Recovered	930 (81.94)	203 (93.55)	1133 (77.17)
	Death	97 (8.55)	4 (1.84)	101 (6.88)
CSF appearance	Clear	441 (38.85)	126 (58.06)	567 (38.62)
	Turbid	519 (45.73)	37 (17.05)	556 (37.87)
	Bloody	42 (3.70)	14 (6.45)	56 (3.81)
	Unknown	50 (4.41)	14 (6.45)	64 (4.35)

**Fig. 1:**
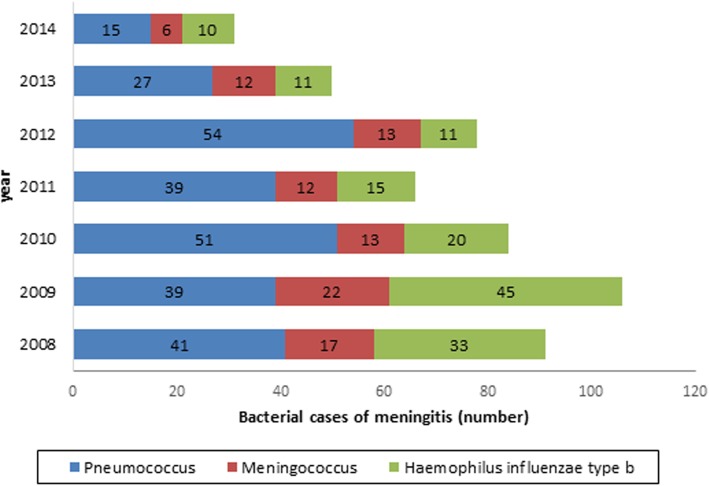
Distribution of bacterial meningitis cases by aetiological agents in Iran, (2008–2014)

The incidence of meningitis due to *Streptococcus pneumoniae* declined during the study period, as its frequency differed from 41 cases in 2008 to 15 cases in 2014. Moreover, *Haemophilus influenzae type b* and *Neisseria meningitidis* also were found to account for 145 (12.78%), 95 (8.37%) cases respectively (p < 0.001). Although, both had a declining trend [Fig. 1]. It should be mentioned that in 629 cases (55.43%) of presumable bacterial meningitis cases in our study, no microorganism was detected. The case-fatality rate (CFR) of bacterial meningitis was 8.55%, with *Neisseria meningitidis* having the highest CFR (18.95%), followed by *Streptococcus pneumoniae* with a CFR of 8.65% while this rate was relatively low for aseptic meningitis (1.84%). In addition, CSF appearance in bacterial meningitis was turbid in 45.73% of cases, clear in 38.85%, bloody in 3.7% and unknown in 4.41%. However, 58.06% of the CSF sample in patients with aspetic meningitis was clear. A comprehensive description of cerebrospinal fluid analysis of patients is provided in [Table 2]. Top three common drug regime for Meningitis cases during study period were ceftriaxone plus vancomycin (46.5%), ceftriaxone (9.4%) and vancomycin (7%).

**Table 2:** Cytological parameters in different type of meningitis in Iran during 2008–2014

Laboratory results	Bacterial CSF	Aseptic CSF
Leukocyte (cell count)	4325.02 (25,977.78)	663.07 (1967.76)
Polymorphonuclear (%)	62.09 (30.60)	43.13 (31.60)
Lymphocytes (%)	34.43 (29.38)	55.97 (33.31)
Proteins (mg/dl)	129.98 (183.20)	73.74 (102.30)
Glucose (mg/dl)	49.39 (42.69)	63.84 (33.54)

More specifically, at the beginning of the study period, during the years between 2008 and 2010, the annual incidence of meningitis of all types had a rising trend (0.28–0.34/100000 population). However, this upward trend ended in the following years until, with some slight fluctuations, it hit a minimum of 0.09 per 100,000 people in 2014 [Table 3].

**Table 3:** Incidence rates of meningitis based on case definition (per 100,000 populations)

Year	Average population	Total cases of meningitis		Confirmed cases of meningitis		Probable cases of meningitis	
		Frequency	Incidence rate	Frequency	Incidence rate	Frequency	Incidence rate
2008	72,266,000	1642	2.27	209	0.28	1433	1.98
2009	73,196,000	1782	2.43	231	0.31	1551	2.11
2010	74,157,000	1587	2.14	257	0.34	1330	1.79
2011	75,150,000	1819	2.42	234	0.31	1585	2.10
2012	76,038,000	1954	2.56	250	0.32	1704	2.24
2013	76,942,000	2100	2.72	216	0.28	1884	2.44
2014	77,856,000	437	0.56	71	0.09	366	0.47

**Fig. 2:**
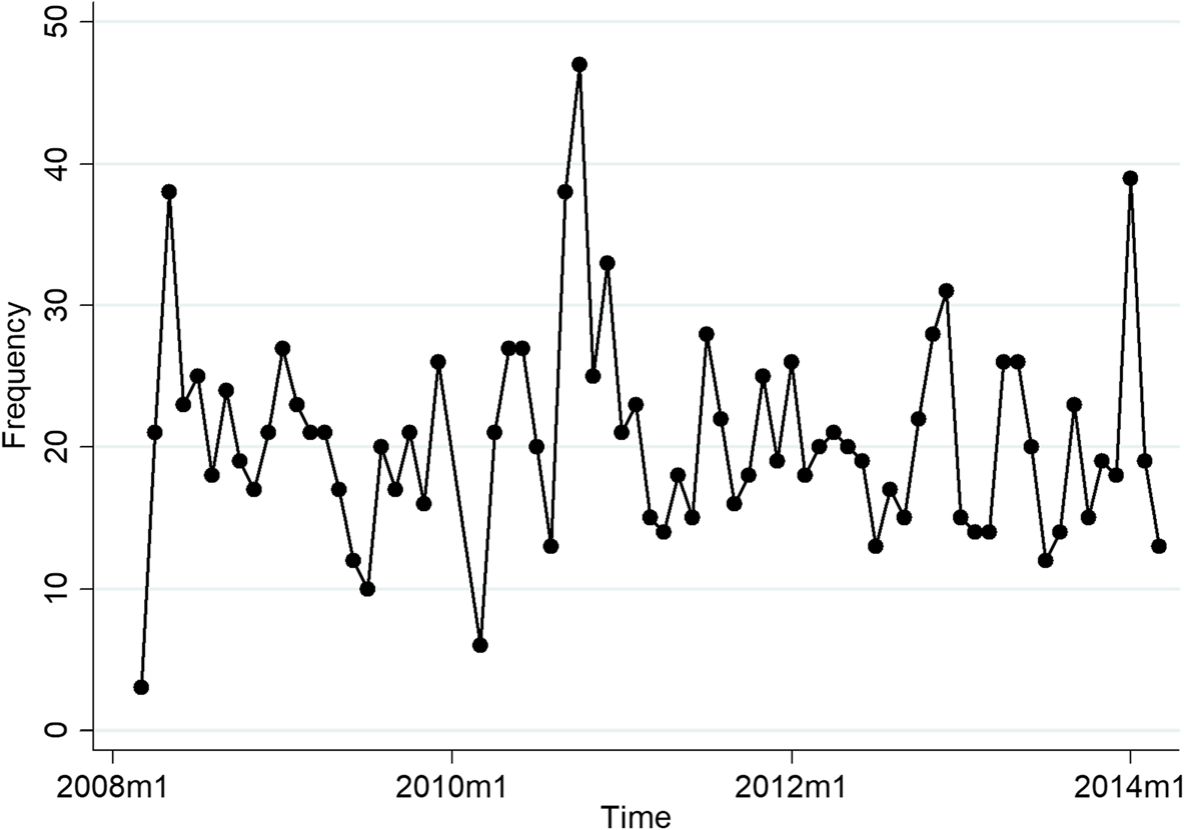
Time trend of monthly counts of meningitis in Iran from March 2008 to December 2014

As for seasonality, as shown in Fig. 2, the results of the present study revealed that the monthly incidence of meningitis showed a wide fluctuation between 47 cases in October 2010 and 3 cases in March 2008. Moreover, the majority of the meningitis cases were reported in cold months from fall to spring [Fig. 2].

## Discussion

Considering the population of Iran in the years under study (March 2008 to December 2014), the estimated annual incidence of meningitis varied from 0.28/100000 population in 2008 to 0.09/100000 population in 2014. However, the published reports showed that meningitis incidence rate in USA was 1.1 cases per 100,000 population [15]. Moreover, the finding of similar studies conducted in several European countries revealed that the incidence rate of bacterial meningitis was 2.6/100000 population in Netherlands, 3.7/100000 population in Italy, 4.3/100000 population in Kozovo and 3.2/100000 population in Iceland [16–19]. In other studies conducted in neighboring countries in the Middle East such as Egypt, Oman and United Arab Emirates, the incidence rate for the meningitis was reported to be 3.65/100000, 3/100000, 1/100000 population, respectively [20–22]. These significant differences in the meningitis incidence rate observed are probably due to different geographical conditions of these countries, immunization status, age groups, time of the study and type of surveillance system prevailing in the countries for the detection of meningitis cases.

Based on the results obtained in the present study, the incidence of meningitis had a downward trend over the years under study. With the same token, the number of cases affected with meningeal pathogen also dropped, a phenomenon which is unrelated to vaccination. In view of the fact that there is currently no countrywide extensive immunization program against the major meningeal pathogen in Iran, with the exclusion of the Hib vaccine which was added into the routine immunization schedule in December of 2014. It must be emphasized that the present study was under way during the year when Hib vaccine was implemented in Iran, the subjects of this study did not receive any vaccines. Therefore, the observed reduction in the incidence rate of meningitis can be accounted for by several other factors including, improved life standards, easier and better access to health care facilities, extensive health education provided for the public, effective preventive and controlling measures taken. However, this observed reduction in incidence rate of meningitis in the country may have been quite artificial, accounted for by the weak surveillance of meningitis, the lack of sufficient attention to surveillance programs of meningitis by the health ministry, high turnover of the personnel unaware of surveillance system, the absence of appropriate infrastructure for the clinical diagnosis of the disease, the lack of transparent rules and regulations for the timely and reliable reporting of cases, a limited laboratory capacity in most regions of the country for definitive diagnosis of early cases with little sophisticated laboratory facilities for isolation pathogens. Contrary to the above observation, the emergence and development of the effective preventive interventions (such as Hib, pneumococcal conjugate vaccines) have dramatically altered the epidemiology of bacterial meningitis in countries where vaccination against the meningeal pathogens is routine. These vaccines not only have significantly reduced the incidence of meningitis caused by these pathogens but also have shifted the median age of the occurrence of bacterial meningitis from infancy to adulthood [23–26].

As mentioned previously, *Streptococcus pneumoniae, Haemophilus influenzae type b, Neisseria meningitidis* are the main triad agents causing bacterial meningitis. Moreover, the most predominant age group unprotected against meningitis is children under the age of 5. Our finding is affirmed by the results of the published works in various geographic regions of Iran [27–29]. However, an observational study in England and Wales noted that *Neisseria meningitidis* was a leading pathogen of bacterial meningitis, followed by *Streptococcus pneumoniae* [30]. On the other hand despite the implementation of immunization program against *Haemophilus influenzae type b*, this organism is still the major cause of bacterial meningitis among young children in Tanzania [31], a finding which is in line with the results obtained in Nepal [32]. *Streptococcus pneumoniae* persists as the foremost organism of bacterial meningitis in the USA. Over the 14 year period (from 1997 to 2010), the remarkable lessening in the incidence rate of meningococcal and H influenza meningitis was observed which is primarily affiliated with the inclusion of conjugated vaccines. At the moment both pathogens have a lower prevalence and have been supplanted by other two common pathogens, that is, Staphylococcus species and Gram-negative bacteria [33]. The spectrum of meningitis pathogens is diverse around the world. It can be caused by one reason or another including, geographical differences, age group, immune function, vaccination coverage, effectiveness of existing vaccines and different immunization situation versus pathogens causing disease.

In this study high rate of meningitis cases presumed to be bacterial without identifying the causative organism. Seventy (4.7%) cases of meningitis with undetermined etiology could be attributable to several factors such as limited facilities of bacteriological culture, the generally low sensitivity of conventional laboratory methods, the lack of the necessary skill and experience of observer for the detection and isolation of pathogens, the failure to give a timely report about disease cases, the use of antibiotic prior to lumbar puncture (LP), all of which can adversely affect the quality of surveillance data. Unfortunately no information concerning the causes of aseptic meningitis is at hand.

Cerebrospinal fluid (CSF) was positive in only 10% of patients, which is consistent with the results of other studies in Iran [34, 35]. However, according to reports published in other countries, CSF culture has been positive in more than 70% cases [36, 37]. The main reasons for such significant difference can be due to the improper use of antibiotic prior to culture, the failure to comply with technical guidelines in storing and transporting samples to the laboratory, incorrect sampling methods and insufficiency of microbiological culturing [38]. In our study approximately 40% of the patients were pre-treated with antibiotics before LP, which can influence test result, leading to negative cerebrospinal fluid culture. Under such circumstances, the use of more advanced method such as antigen detection and PCR seems advisable and useful.

Male ascendancy in meningitis was discovered in the present study which is in keeping with the other ones. It can be attributable to biological or social factors [39, 40].

In the present study, the CSF analysis demonstrated that the median value of leukocytes count in patients with bacterial meningitis was 4325 cell/mm3, with polymorphonuclear (PMN) predominance which is persistent with previous studies [41, 42]. However, this value was reported 663 cell/mm with lymphocytes dominance in aseptic meningitis including viral cases. Considering the fact that a cell count lower than 100 is more prevalent in viral meningitis [43], our result are in conflict with those found by Wang et al. [44]. This inflated rise can be due to peripheral blood in CSF sample following LP and LP technique [43].

In this study, the median of CSF glucose concentration was reported 49.39 mg/dl in bacterial meningitis, which is different than results of other studies [45]. The CSF glucose level is used as a basis to distinguish bacterial meningitis (in which it usually decreased, usually < 40 mg/dl) from viral meningitis (in which the glucose levels are usually unaltered). Generally, A CSF: blood glucose ratio of less than 0.4 shows hypoglycorrhachia [46]. However, in our survey, the median CSF glucose in patients was higher than 40 mg/dl. This observed increase may have been caused by the administration of antibiotics before hospitalization, and the LP performance. Under such conditions, bacteria can be killed and consequently, the consumption of glucose reduces [47]. As CSF glucose is about two third of the serum glucose, an increase in the level of serum glucose can trigger an increase in of CSF glucose level [43]. CSF Glucose level is normal in viral meningitis. Another diagnostic indicator for distinguishing bacterial from viral meningitis is CSF protein values. In this study, the median value of protein was higher in bacterial meningitis than viral meningitis, which is similar to those of other studies [48, 49].

A retrospective design contingent upon surveillance data is imperfect, inaccurate and less important compared to the one which uses prospective data. However, it should be kept in mind that such data are not currently accessible.

In this study, the estimated incidence rate of meningitis during the time-span under study did not reflect the actual burden of meningitis in Iran primarily because the analyses were conducted only on laboratory-tested cases of meningitis (culture positive). Due to the unavailability of more advanced diagnostic evaluations (such as PCR), the identification of culture-negative cases in the laboratory across the country is not possible; the present study did not contain such data. Underestimation of incidence of meningitis because of passive approach of national notifiable diseases surveillance system of meningitis in Iran and lack of appropriate data on serotype distribution of meningitis cases are other limitation of this work.

Moreover, the currently operating surveillance does not provide the comprehensive information concerning some epidemiologic features of meningitis for reported cases, serotype distribution and replacement in this study as highlighted by published literature [50, 51]. Nor were there information on causative agents for viral meningitis, serotypes distribution of bacterial meningitis agents, antimicrobial resistance and clinical outcome of patients. Even information published on a series of meningitis variables is not completely represented. Finally, high rate of cases with undetermined pathogens is another major shortcoming of our study.

Despite the limitations mentioned, the present study is the first in its type in that the analyses were conducted on a representative collection of patients with meningitis from all over the country. Therefore, the findings obtained enjoy a good generalizability characteristic; with direct implications for of study results and the obtained output from this survey can be helpful in identifying the main priorities for development of efficient control and prevention programs of meningitis. In addition, the results of this study can be used to revising the surveillance system of meningitis, monitoring the trend of invasive pneumococcal disease and decision to PCV introduction.

## Conclusions

Unlike previous studies, the findings of the present study showed that bacterial meningitis was more common than aseptic meningitis. Rate of culture positivity was very low. Similar to studies in other countries, the incidence rate of meningitis decreased during the study period in Iran, with no convincing and plausible explanation specified for the observed reduction in incidence rate. Nonetheless, it should be noted that reported confirmed cases of meningitis by national notifiable diseases surveillance system of meningitis has limited to January, February and March 2014. This may leaded to observe such decline in the incidence rate of meningitis during 2014 in comparison to previous years of study period.

*Streptococcus pneumoniae, Haemophilus influenzae type b* and *Neisseria meningitidis* were the three most prevailing causes of bacterial meningitis which affects more children under the age of 5. Some indicators derived from the CSF analysis such as PMN percentage, the CSF glucose and protein values were used as bases for the differentiation of aseptic from bacterial meningitis. The findings of this study emphasize the need for more efficient surveillance and control of meningitis in Iran under the light of following recommendations:

- Continuous monitoring the national notifiable diseases surveillance system of and implementing an enhanced surveillance system
- Establishment of the strict and precise rules and regulation for the timely reporting of cases
- Applying experienced and well-trained human resources and making optimal use of available human resources

- Enhancing and empowering laboratory capacity throughout the country for definitive diagnosis of cases along with sophisticated laboratory facilities.

## Data Availability

The dataset of the current study is available from the corresponding author at a reasonable request.

## Abbreviations

CFR: Case fatality rate
CSF: Cerebrospinal fluid
DALY: Disability-adjusted life year
PCV: Pneumococcal conjugate vaccine
